# Impact of hospital mergers: a systematic review focusing on healthcare quality measures

**DOI:** 10.1093/eurpub/ckac002

**Published:** 2022-02-14

**Authors:** Marco Mariani, Leuconoe Grazia Sisti, Claudia Isonne, Angelo Nardi, Rosario Mete, Walter Ricciardi, Paolo Villari, Corrado De Vito, Gianfranco Damiani

**Affiliations:** 1 Sezione di Igiene, Dipartimento Universitario Scienze della Vita e Sanità Pubblica, Università Cattolica del Sacro Cuore, Roma, Italia; 2 Istituto Nazionale per la promozione della salute delle popolazioni migranti ed il contrasto delle malattie della Povertà (INMP), Roma, Italia; 3 Center for Global Health Research and Studies, Università Cattolica del Sacro Cuore, Roma, Italia; 4 Sapienza Università di Roma, Roma, Italia; 5 Fondazione Policlinico Universitario A. Gemelli IRCCS, Roma, Italia

## Abstract

**Background:**

Despite mergers have increasingly affected hospitals in the recent decades, literature on the impact of hospitals mergers on healthcare quality measures (HQM) is still lacking. Our research aimed to systematically review evidence regarding the impact of hospital mergers on HQM focusing especially on process indicators and clinical outcomes.

**Methods:**

The search was carried out until January 2020 using the *Population, Intervention, Comparison and Outcome* model, querying electronic databases (MEDLINE, Scopus, Web Of Science) and refining the search with hand search. Studies that assessed HQM of hospitals that have undergone a merger were included. HQMs were analyzed through a narrative synthesis and a strength of the evidence analysis based on the quality of the studies and the consistency of the findings.

**Results:**

The 16 articles, included in the narrative synthesis, reported inconsistent findings and few statistically significant results. All indicators analyzed showed an insufficient strength of evidence to achieve conclusive results. However, a tendency in the decrease of the number of beds, hospital staff and inpatient admissions and an increase in both mortality and readmission rate for acute myocardial infarction and stroke emerged in our analysis.

**Conclusions:**

In our study, there is no strong evidence of improvement or worsening of HQM in hospital mergers. Since a limited amount of studies currently exists, additional studies are needed. In the meanwhile, hospital managers involved in mergers should adopt a clear evaluation framework with indicators that help to periodically and systematically assess HQM ascertaining that mergers ensure and primarily do not reduce the quality of care.

## Introduction

Over the last three decades, a growing number of healthcare organizations of different countries, especially in the USA and in Europe, underwent mergers.^1–3^ Referring to hospitals, the merger is defined as a combination of previously independent hospitals, formed by either the dissolution of one hospital and its absorption by another, or the creation of a new hospital from the dissolution of all participating hospitals.^4^ Hospitals mergers might have been encouraged by some national policies such as the introduction of the *Medicare Prospective Payment System* and the growth of the managed care in the 1980s in the USA^5^^,^^6^ and the promotion of competition inside the health system market promoted by the Conservative Government since 1991 in the UK.^2^ These merger processes have determined substantial changes in healthcare markets.^2^ In the USA, in 1994, more than 10% of the hospitals resulted as involved in some form of mergers^6^ reaching a peak of 2,497 mergers in 2003.^6^ Similarly, in the UK, between 1997 and 2006 more than 100 mergers were started; in the early 2000s, there were 180 acute National Health Service (NHS) trusts while, by 2015, the number of acute foundation trusts and NHS trusts had dropped to 150 (17% reduction).^2^^,^^7^ In Italy, in recent years, the merger policy led to a 30% reduction in the number of hospitals: from 142 hospitals in 2011 to 99 in 2018^8^ and also in Northern Europe, countries as Norway and Denmark undertook similar processes.^9^^,^^10^

Drivers for mergers are different and depend on countries and health system features: in Italy and in the UK, mergers were primarily driven by political decisions, while in USA, where federal or state government has less power to set the policy agenda, they were mainly entrepreneurial and market-driven.^2^^,^^11^ In literature, political and economic issues are reported as the main merger drivers.  Aimed at achieving economies of scale and scope, rationalizing services, financially sustaining smaller hospitals, increasing the ‘bargaining power’ and enlarging organizations to address commissioner challenges.^12^^,^^13^ 

Affecting the catchment area, the hospital organization and functioning and increasing the market concentration, hospital mergers could potentially impact healthcare quality measures (HQMs) affecting patients as well as employees and communities. In line with the volume–outcome relationship, service consolidation could improve the quality of care, for example with regards to surgical procedures. Nevertheless, some studies argued that less concentrated and high competitive markets report better clinical outcomes in hospitalized patients, i.e. in terms of mortality.^14^^,^^15^ Conversely, clinical outcomes seem to remain unchanged or rather worsened among acquired or merged hospitals.^16^

Despite this, to date, literature on the impact of hospitals mergers appears to have mainly focused on economic aspects.^17^ On the other hand, to the best of our knowledge, literature summarizing the impact of merger on HQM is still lacking. Consequently, we aimed at systematically review available evidence regarding the impact of hospital mergers on HQM focusing especially on processes indicators and clinical outcomes.

## Methods

### Literature search and study selection

A systematic research was conducted querying MEDLINE, Scopus, Web of Science from their commencement until January 2020. To gather evidence on the impact of hospital merger on HQM, a search string was elaborated using the *Population, Intervention, Comparison, Outcome* (PICO) model.^18^ In detail, we identified the ‘population’ as hospital facilities, the intervention as the merger, the comparator as non-merged hospital or the same hospital in the pre-merger status, and the outcome as structure, process and outcome HQM.^19^ Search strings were built using the following keywords: ‘hospital’, ‘merger’, ‘consolidation’, ‘outcome’, ‘process’, ‘indicators’, ‘performance’, ‘measure’ (Supplementary data). When available, the use of MeSH terms allowed us to retrieve more comprehensive information. The search was refined by hand search performed using Google Scholar, analysis of bibliographic citations and experts recommendation. The research was carried out using the Preferred Reporting Items for Systematic Reviews and Meta-Analyses (PRISMA) guiding the systematic review reporting and was restricted to articles published in English.^20^

Studies were considered eligible if they quantitatively assessed HQM of hospitals that have undergone a merger. In case of perception/satisfaction of employees qualitative studies were screened for inclusion as well. Exclusion criteria were represented by publications without original data (e.g. reviews, editorials) and with intervention represented by vertical merger or by non-homogeneous mergers such as those between hospital and non-hospital facilities. Regarding structure healthcare measures, financial indicators were not included because previously addressed in literature.^17^ Two investigators independently screened titles and abstracts of all records and evaluated full texts of potentially eligible studies for their inclusion in the review. At all levels, disagreement and discrepancies between the investigators were solved by consensus.

### Data extraction

From each study, the following data were extracted: first author's last name, year of publication, country, study design, period of observation (before and after merger), number of hospital involved in the merger, main investigated outcomes, main results in HQM classified in structure, process and outcome.^21^

### Quality assessment

The risk of bias (RoB) and the methodological quality of the included studies were assessed using the National Institutes od Health (NIH) Study Quality Assessment tools for before–after and case–control studies, and The Joanna Briggs Institute Systematic Reviews Checklist for qualitative researches.^22^^,^^23^ NIH Study Quality Assessment tools include items regarding study objective and population, sample size, inclusion–exclusion criteria, blinding of outcome assessor, and appropriate statistical analysis. Furthermore, NIH tools encompass control group selection criteria and exposure factor items for case–control studies, and attrition rate and outcome measures items for before–after studies.^22^ According to The Joanna Briggs Institute Systematic Reviews Checklist,^23^ congruity among philosophical perspective, research methodology, objectives, methods used to collect and analyze data, interpretation of results and researcher influence on the study were described and assessed for qualitative research. Any disagreement in RoB evaluation was solved by discussion and, if necessary, a third reviewer was involved. Based on the reported scale, articles were classified into three levels of RoB: low (76–100%), moderate (26–75%) or high (0–25%).

### Data synthesis

Studies included showed methodological heterogeneity, particularly regarding study design and outcomes reporting. Few studies considered similar outcomes, and when they did, they had either different control group or different methodology of outcome assessment. A pooled analysis would have limited utility, therefore, a meta-analysis was deemed inappropriate. We preferred to synthesize data through a narrative synthesis of the study findings focusing on outcomes of interest reporting statistically significant results.

To further support the narrative synthesis and easier the identification of consistent results (i.e. increasing or decreasing of the same indicator in different studies), we performed a strength of evidence analysis according to literature.^24^^,^^25^

The strength of the evidence was performed using a rating system based on the methodological quality of the studies and the consistency of results.^24^^,^^25^ Only outcomes reported in two or more studies were evaluated. Results were synthesized into three levels of scientific evidence^25^:

- strong evidence: provided by generally consistent findings in multiple high-quality studies;
- moderate evidence: provided by generally consistent findings, in one high-quality study and one or more moderate-quality studies or in multiple moderate-quality studies; and
- insufficient evidence: inconsistent findings in multiple studies.

Study findings were considered consistent if >75% of the studies reported the same conclusion with statistically significant results (*P* < 0.05).^24^^,^^25^ High- and moderate-quality studies were identified through the RoB assessment. In case of insufficient evidence, a sensitivity analysis rating the outcome as promising and not promising was also performed. Outcomes were considered as *promising,* in presence of at least two significance in outcomes and >75% of consistent findings.

## Results

### Study selection

**Figure 1:**
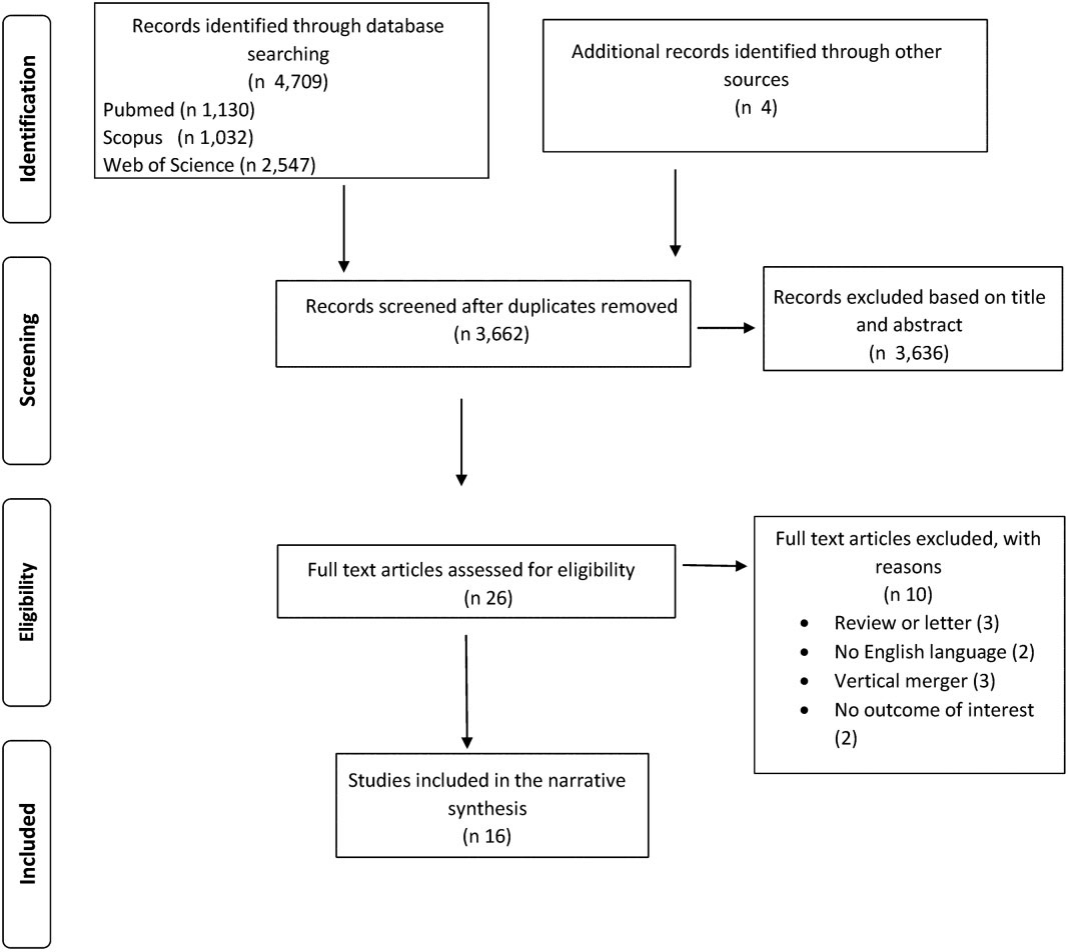
PRISMA flowchart of studies selection

The results of abstract and full-text screening with reasons for exclusion are shown in the PRISMA diagram^20^ in figure 1. The database research resulted in 4,709 records while 4 articles were retrieved through hand search. After checking for duplicates, 3,662 articles were analyzed for eligibility and 3,636 were excluded after the screening of titles and abstracts. The remaining 26 articles were selected for full-text review resulting in 16 articles eventually included in the analysis.

### Data extraction

Characteristics of the studies included in the analysis are reported in table 1. Years of publication of the studies ranged from 1997 of Alexander et al.^4^ to 2020 of Beaulieu et al.^26^ A total of 62.5% of the included articles were longitudinal retrospective before–after studies, of which 40% were controlled studies and the other 50% cross-sectional studies, only one study included both a cross-sectional (survey) and a before–after design. The majority (56.3%) were developed in the USA, followed by Northern Europe (Norway, Denmark, Sweden) accounting for 31.3% of all studies. The sample size of hospitals involved in the merger ranged from 246 hospitals^26^ to the merger of only 2 hospitals into 1.^27^^,^^28^ The period of observation, when specified, ranged from 3 months to 8 years with a mean of 2 years before the merger and of 3 years after.

**Table 1:** Studies characteristics

References	Country	Study design	Study period (years if not otherwise specified)			Number of merged hospitals	Outcomes
			Total	Before	After		
Alexander et al.^4^	USA	Controlled before–after	8	–	–	194 in 97	Average of operational beds and of adjusted admissions; occupancy rates per adjusted admissions; total number of personnel; total number of nurses
Beaulieu et al.^26^	USA	Controlled before–after	8	2 or 3	3 or 4	246 in 198	Patient-experience composite indicator (five items from the Hospital Consumer Assessment of Healthcare Providers), clinical process composite indicator (seven measures of cardiac, pneumonia and perioperative care), 30-day readmission rate, 30-day mortality rate
Christiansen et al.^9^	Denmark	Before–after	9	–	–	40 in 21	Number of doctors, number of nurses, number of other healthcare personnel, number of social and healthcare assistants, number of total employed full-time employees (FTEs) number of beds, number of admissions, number of ambulatory visits, number of ambulatory patients, length of stay, percentage of eligible day surgery actually performed, number of surgery patients, waiting time for planned surgery
Dranove et al.^5^	USA	Controlled before–after	5	1	4	–	Number of inpatient admissions, number of outpatient visits, number of SNF admissions, number of ER visits, percentage of births, case mix index
Engström et al.^27^	Sweden	Cross-sectional study (semi-structured interviews)	6 months	1 months	5 months	2 in 1	Perception of personnel (31 interviews: 10 nurses and 8 managers, others: physicians, support staff, secretaries, practical nurses)
Gaynor et al.^2^	USA	Before–after	6	2	4	223 in 112	Number of beds, number of total staff, number of total admissions, percentage staff that are med. qualified, percentage of staff management expert, percentage of experts on agency staff, time waited for admission; mean length of stay, mean waited time, percentage of list that waited >180 days, AMI death rate within 30 days of discharge, stroke death rate within 30 day of discharge, 28-day readmission rate for stroke, 50-day return rate for stroke, 28-day readmission rate for FPF, 28-day return rate for FPF
Harris et al.^30^	USA	Before–after	5	–	–	41 in 20	Number of outpatient visits; number of adjusted discharges; number of diagnostic and special services (service mix), number of operational beds, number of employees (non-physician FTEs and half-of part-time workers employed)
Hayford et al.^16^	USA	Before –after	16	–	–	40 (NA)	Percentage of patients received Intensive heart surgery (bypass surgery or angioplasty); percentage of them treated within one day, average number of procedures, inpatient mortality, average length of stay for IHD, average number of ischaemic discharges
Ho et al.^31^	USA	Before–after	6	from 1 to 5	from 1 to 5	21 (NA)	Inpatient mortality for heart attack and stroke patients, 90-day readmission rate for heart attack patients, discharge within 48 h for normal newborn babies
Holm-Petersen et al.^35^	Denmark	Cross sectional study (semi-structured interviews)	3	0	3	NA	Perception of personnel (103 interviews in groups of nurse staff, practical nurses and nurse leaders) plus senior doctors and middle management interviews
Ingebrigtsen et al.^29^	Norway	Before–after, and cross sectional study	5.8	5	4 months	3 in 1	Waiting time, number of patient visits, satisfaction of personnel (3119 employees)
Noether et al.^32^	USA	Cross-sectional study (structured interviews)	–	–	–	–	Perception of personnel (20 hospital executives)
Roald et al.^34^	Norway	Cross-sectional study (semi-structured interviews)	–	–	–	2 in 1	Perception of personnel (14 informants)
Romano et al.^28^	USA	Controlled before–after	5	2	3^b^	2 in 1	CABG and PCI mortality, CHF, pneumonia and stroke mortality, AMI mortality; number of decubitis ulcers, number of FTR, number of selected infections due to medical care and post-operative hip fracture, birth trauma, obstetric trauma, neonatal mortality
Shaw et al.^36^	UK	Cross-sectional study (semi-structured interviews)	3 months	0	3 months	2 in 1	Perception of personnel (42 interviews: senior trust managers and professional staff)
Staňková et al.^33^	Czech Republic	Cross-sectional study (structured interviews—close ended questions)	5 months	–	–	15 (NA)	Perception of personnel (15 hospital directors)

CABG: coronary artery bypass grafting; CHF: chronic heart failure; ER: emergency room; FPF: fractured proximal femur; FTE: full time equivalent; FTR: failure to rescue (death among surgical patients with potentially serious but treatable in-hospital complications); IHD: ischaemic heart disease; IT: information technology; PCI: percutaneous coronary intervention; SNF: skilled nursing facilities.

For Romano et al.,^28^ main outcome obstetric trauma both vaginal with and without instrument are considered. For perception in staff outcome: in Holm-Peterson et al.,^35^ satisfaction, leadership tasks, delegation, reflections on size of hospital wards are explored; Cost-reduction benefits, improvement of clinical quality, ability to assume payment risk dimensions are analyzed in Noether et al.^32^

a Results in logaritmic scale;

b year of merger excluded;

c DID: difference in difference.

Regarding HQM, the majority of the studies focused on process measures with 93.8%^2^^,^^4^^,^^5^^,^^9^^,^^16^^,^^26^^,^^27^^,^^29–36^ of them reporting at least one process measure. Six studies evaluated only the perception of staff concerning the merger process^27^^,^^32–36^; three (18.7%) analyzed both process and structure measures^4^^,^^9^^,^^30^; three (18.8%)^16^^,^^26^^,^^31^ process and outcome measures, eventually only one study^2^ has analyzed indicators belonging to all dimensions of healthcare quality (structure, process and outcome).

In detail, regarding the structure measures, 25% of the included studies reported the number of beds and measures related to the staff (overall or stratified according to the professional role).^2^^,^^4^^,^^9^^,^^30^ Regarding process measures, the majority of HQM investigated were: waiting time for admission, number of visits, length of stay and perception of staff. As to this latter, 57.1% of studies were represented by semi-structured interviews and 28.6% by structured interviews,^32^^,^^33^ in only one case^29^ an unspecified survey was used. The sample sizes for personnel interviews ranged from 14^34^ to 3,119^29^ informants. Personnel interviewed were mainly represented by different healthcare professional staff (71.4%), while two studies^32^^,^^33^ specifically focused on chief executives.

Outcome measures were reported in five studies^2^^,^^16^^,^^26^^,^^28^^,^^31^ and the mostly represented measures were related to: overall hospital mortality rate (40%), acute myocardial infarction (AMI) mortality (60%) and stroke mortality (60%).

### Risk of bias

An overall judgement of the included studies is available online (Supplementary data, table S1). The quality assessment showed variability in the overall RoB, from moderate to low. Low RoB was reported: in all case–control studies, in 16.7% of six qualitative researches and in 70% of before–after studies. Ethical approval and researchers’ cultural or theoretical orientation were not reported in all of qualitative researches; only one^35^ qualitative study considered researcher influence on the research. Three before–after studies used interrupted time-series design.^4^^,^^26^^,^^29^

### Data synthesis

The included studies showed methodological heterogeneity in terms of statistical analysis: five adopted difference-in-differences analysis to compare changes in outcomes in merging hospitals to changes in a group of hospital controls not involved in a merger^2^^,^^4^^,^^5^^,^^26^^,^^28^; two studies^16^^,^^31^ built regression models to account for confounding variables, and in one^30^ it was possible to extract only the *t*-test values for the indicators analyzed before and after the hospital merger. To facilitate the synthesis of significant findings, table 2 provides the studies’ main statistical significant results of healthcare quality measures classified into structure, process and clinical outcomes indicators.

**Table 2:** Main statistically significant results of single studies

References	Main statistical significant results		
	Structure	Process	Outcome
Alexander et al.^4^	Decrease: average of operational beds (DID)^c^ (−33.98)	Increase: occupancy rates (DID)^c^ (+3.41) Decrease: average of adjusted admissions (DID)^c^ (−743.47)	–
Beaulieu et al.^26^	–	Increase: clinical process composite indicator (+0.2 standard deviation)	Decrease: patient-experience composite indicator (−0.17 standard deviation)
Christiansen et al.^9^	Increase: n. of doctors (19.25%), number of nurses (13.19%) and other healthcare professionals (19.14%) Decrease: number of social and healthcare assistants (16.74%)	Increase: ambulatory visits (+32.32%) Decrease: waiting time (−28.79%), length of stay (−20.51%)	–
Dranove et al.^5^	–	Decrease: no. of admissions (–6289.35) (DID)^c^; outpatient visits (−28231.28) (DID)^c^, ER visits (−6111. 15) (DID)^c^	–
Engström et al.^27^	–	Low degree of involvement, commitment and communication in merger process, lack of trust in managers competence to lead and manage the merger, lack of trust in politician competence and vision, no adequate coordination between hospitals, lack of communication to citizens, opportunities for distributing resources adequately and for professional growth	–
Gaynor et al.^2^	Decrease: (log linear time trend) total number of staff (−0.12)^a^, total number of beds (−0.12)^a^	Increase: mean time waited per admission (+0.10)^a^ and share of patients waited 180 days or more (+3.22)^a^ Decrease: total admissions (−0.11)^a^	–
Harris et al.^30^	–	–	–
Hayford et al.^16^	–	Increase: 2% more average number of procedure (coefficient 0.12); utilization of intensive heart surgeries (coefficient 0.05)	Increase: <1% increase average length of stay
Ho et al.^31^	–	Increase: 90-day readmission for heart attack (+1.7%)	–
Holm-Petersen et al.^35^	–	Nurses stress a disorganized leadership: distance to their leader, goals and direction unclear, nurse leaders and middle management stress difficulties to manage and communicate with a large staff. ‘No follow-up’, ‘not being seen’, ‘role overload’, but they also report more development possibilities and ‘greater flexibility’	–
Ingebrigtsen et al.^29^	–	81% employees satisfied after merging	–
Noether et al.^32^	–	Saving in fixed costs especially associated with supply chain, IT, administration, billings, pharmacy and laboratory and physical plant management. Improvement of hospital quality. Need to long-term commitment, organizational change and consolidation of hospital services and cultural change	–
Roald et al.^34^	–	Goal uncertainty and distance between decision makers and employees, strong differences in culture of the two hospitals and fear to be ruled by the other hospital, individual insecurity of professional positions reached	–
Romano et al.^28^	–	–	Increase: AMI mortality (AHRQ): EH (+4.96); Pneumonia mortality: EH (+3.14); Stroke mortality: EH (+4.94); Post-operative hip fracture: EH (+0.09); Birth trauma: HPH (+0.33%) EH (+ 0.74%); Neonatal mortality EH (+0.32); Decrease: Obstetric trauma HPH (−1.14%) EH (−1.08%); Decubitis ulcers HPH (−0.76%) EH (−0.56%), Selected infections due to medical care: EH (−0.05%)
Shaw et al.^36^	–	Cultural differences between hospital merged. Uncertainty for the future, vacant posts, double work-loads, difficulties in contacting leaders and access information, opportunity for personal and professional growth	–
Staňková et al.^33^	–	Integration perceived as an advantage (66.7%) with better negotiations with suppliers (93%), health companies (80%), and cost reduction (73%). Disadvantages seen especially in more complex change promotion (73%) and communication (60%), decreased autonomy, increased administration burden (especially in short term)	–

For Romano et al.,^28^ EH is the acquiring hospital, HPH the acquired hospital. In Obstetric Trauma both vaginal with and without instrument are considered. For perception in staff outcome: in Holm Peterson et al.,^35^ satisfaction, leadership tasks, delegation, reflections on size of hospital wards are explored; cost-reduction benefits, improvement of clinical quality, ability to assume payment risk dimensions are analyzed in Noether et al.^32^

a Results in logaritmic scale;

b year of merger excluded;

c DID: difference in difference.

AHRQ: Agency for Healthcare Research and Quality; EH: Evanston Northwestern Hospital; ER: Emergency Room; HPH: Highland Park Hospital; IT: Information Technology.

### Structure indicators

Concerning structure indicators, the change in number of beds after merger showed a decreasing trend: statistically significant decreases in two studies (−0.12 on logarithmic scale; −33.98)^2^^,^^4^ and a non-statistical significant decrease in another study (−20.7, *t*-value 0.35) were found.^29^ Three studies evaluated change in hospital staff number: one^2^ (table 2) showing a significant decrease in total hospital staff (−0.12 on logarithmic scale), one^9^ showing a non-significant reduction in the number of physician employees (−19, *t*-value 0.05), and the other^4^ a non-significant negative value in the difference-in-difference analysis for total personnel per average daily (−35.68).

### Process indicators

Regarding process indicators, three studies^2^^,^^4^^,^^5^ showed a statistically significant decrease in inpatient admission (−0.11 in logarithmic scale; −743,47; −6289.35) (table 2). Two studies evaluated changes in the number of outpatient visits, one with a non-significant increase^30^ (2750, *t*-value 0.07) while the other^5^ showed a statistically significant negative value in the difference-in-differences analysis (−28231.28). Acquired hospitals showed, in one study^26^, an improvement in the clinical process composite measure (0.22 standard deviation; *P* = 0.03) but probably not attributable to the merger since the improvement started before the acquisition.

Regarding personnel satisfaction, hospital executives considered mergers positively, especially for increased negotiation skills and costs reduction. Conversely, personnel expressed criticalities mainly regarding the different organization of merged hospitals, the inadequate communication of the merger process, the uncertainty of job positions and the lack of personnel involvement in the post-merger phase.

### Clinical outcome indicators

HQM that were mostly assessed in the studies included those related to AMI mortality, stroke mortality and hospital readmission. Only one study assessing AMI mortality showed a significant negative effect on the outcome^28^ finding a statistically significant increase of 4.46% in one of the hospitals that merged (the acquiring hospital). Among the four studies^2^^,^^16^^,^^28^^,^^31^ assessing AMI mortality, three presented a non-significant worsening effect: one reported a coefficient of 0.003^16^; the acquired hospital in another study showed a difference of + 2.22%^28^; another a coefficient of 0.022^31^ and, eventually, the last one^2^ a non-significant positive effect on mortality (−0.004 in logarithmic scale).

Regarding stroke mortality, one study^28^ found a significant negative effect after merger (showing an increase of +4.94% for the acquiring hospital) (table 2); two studies^2^^,^^28^ showed that the negative effect of the merger was non-significant (one^2^ 0.055 in logarithmic scale; the other^28^ +2.42%); on the other hand, eventually, a non-statistically positive effect was found in one study.^31^ Of the three studies that evaluated hospital readmissions, one^31^ showed a negative effect of merger, with a statistically significant increase of the 90-day readmission for heart attack, while the other two showed a non-statistical significant positive value of: 28-day stroke readmission rate^2^ and 30-day readmission rate (−0.10 percentage points; 95% confidence interval −0.53 - +0.34; *P*.72).^26^

### Strength of evidence

The strength of the evidence rating system resulted as “insufficient” for all considered indicators. The sensitivity analysis did not show promising results with the exception of the decrease in the number of beds and in the inpatient admissions (table 3).

**Table 3:** Results of the strength of evidence

Main findings	References	Direction of results	Strength of evidence	Sensitivity analysis^a^
Structure indicators				
Beds			Insufficient	Promising
	Gaynor et al.^2^	–		
	Alexander et al.^4^	–		
	Harris et al.^30^	NS (−)		
Staff			Insufficient	Not promising
Total medical and non-medical staff	Gaynor et al.^2^	–		
Total personnel per average daily	Alexander et al.^4^	NS (−)		
Non-physician FTEs plus half of the part-time workers	Harris et al.^30^	NS (−)		
Process indicators				
Inpatient admissions			Insufficient	Promising
	Dranove et al.^5^	–		
	Gaynor et al.^2^	–		
	Alexander et al.^4^	–		
Outpatient visits			Insufficient	Not promising
	Dranove et al.^5^	–		
	Harris et al.^30^	NS (+)		
Outcome indicators				
AMI mortality			Insufficient	Not promising
30-day mortality on or after discharge per AMI	Gaynor et al.^2^	NS (−)		
Inpatient heart attack	Ho et al.^31^	NS (+)		
HPH AMI mortality AHRQ	Romano et al.^28^	NS (+)		
EH AMI mortality AHRQ	Romano et al.^28^	+		
	Hayford et al.^16^	NS (+)		
Stroke mortality			Insufficient	Not promising
30-day mortality on or after discharge per stroke	Gaynor et al.^2^	NS (+)		
Inpatient stroke	Ho et al.^31^	NS (−)		
EH stroke mortality	Romano et al.^28^	+		
HPH stroke mortality	Romano et al.^28^	NS (+)		
Readmissions			Insufficient	Not promising
28-day stroke readmission rate	Gaynor et al.^2^	NS (+)		
90-day heart attack	Ho et al.^31^	+		
30-day readmission rate	Beaulieu et al.^26^	NS (−)		

a Promising: at least two significance in outcomes and >75% of consistent findings.

NS, non-significant; +: statistical significant increase of outcome in merged hospitals; −: statistical significant decrease of outcome in merged hospitals; EH: Evanston Northwestern Hospital; FTE: full time equivalent; HPH: Highland Park Hospital; AHRQ: Agency for Healthcare Research and Quality Indicator.

## Discussion

In literature, the main potential benefits of mergers declared by hospital leaders usually refer to economic and financial availability, but also clinical quality improvements due to increased investments, higher volumes of specialized procedures and standardization of clinical protocols.^32^

Studies analyzed in our research show that, apart from some structural and process indicators, hospital mergers resulted in non-significant improvement in the HQM compared to the period before the merger itself.

Regarding structural and process indicators, the reduction in the number of beds and in inpatients admissions (table 3) could be related to a structural and functional remodelling of the merged hospitals that seek to pursue economies of scale setting the number of beds at 200–300 and keeping the annual discharges at less than 10,000.^1^^,^^17^ Nonetheless, the tendency towards an operational reduction could depend on the pre-merger phase, especially for hospitals that merge due to financial constraints. The trend towards a reduction of the number of staff could be seen in the light of gaining efficiency by increasing hospital productivity.

Respect to the volume–clinical outcome relation, it is reported to widely vary across conditions and outcomes, with the largest benefits occurring among a small number of technically difficult surgical interventions.^17^ Studies included in our research did not reach sufficient strength of evidence towards a clear improvement or worsening; only one study^28^ showed a statistically significant worsening in relation to stroke and AMI mortality. Nonetheless, a tendency towards a worsening includes also other clinical outcomes such as stroke mortality and readmissions.

Lack of evidence of improved clinical quality after a merger is in line with a new research^26^ that highlights that mergers do not produce significant differential change in 30-day readmission rates or in 30-day mortality and does find inconclusive improvement in clinical process measures. In addition, this study underlines that hospital mergers are associated with modest but significant deterioration in patients’ experiences. Similar findings are reported by the American Hospital Association in a study^32^ that stressed that, apart from the substantial quality benefits noted by hospital leaders, small positive improvements are reported, above all in readmission rates.

Overall, the effects of the mergers could depend on some features—as the type of the organizations involved—and the merger between similar organizations, sharing the same culture and organizational attitude, appears to be more effective.^17^ The early involvement and engagement of hospital staff in the pre-merger phase followed by their proper involvement over time^26^ could also help to ensure the success of the merger, allowing both the structural and the functional integration of the hospitals. This involvement could limit the disadvantages of the organizational change that could undermine clinical outcomes, especially in the short time.^31^

This systematic review represents a first attempt to summarize evidence regarding the impact of hospital mergers on HQM with the purpose of providing a broader picture regarding research and practice on hospital merging processes.

The strengths of this review include an *a priori* methodology with an accurate search strategy involving different electronic databases supplemented by hand searching, forward citation searching, study identification, appraisal, data extraction and description. Furthermore, we sought to increase the value and the validity and reliability of findings performing strength of evidence analysis. Nonetheless, several limitations could be identified. First, our research has included only publicly available English-written articles; therefore, a language bias cannot be excluded. We cannot rule out that the wide variability in the terms used to define merger, as well as the national and governmental dimension of the topic, might have limited the inclusiveness of our research.

Most of the included studies provided only descriptive statistics and some outcomes (benefits or drawbacks) achieved after merging hospitals had been evaluated only in single studies consequently providing little inferential and generalizable information.

High heterogeneity in the studies’ methodology was found, e.g. in measures used to evaluate results and in the follow-up duration (from months to years), hindering undertaking a formal meta-analysis and performing a rigorous strength of evidence analysis.

Studies included in our analysis do not show strong evidence that hospital mergers impact HQM by bringing net improvements. At the moment, few studies indicate that the benefits related to mergers could be met counteracting the potential unintended consequences as decreased competition, higher prices for patients and reduced geographic coverage of services that mergers could cause.

Since a limited amount of studies currently exists, there is a need for additional studies providing concrete evidence that hospital mergers ensure and primarily do not reduce high-quality care that patients require. Researchers should work to produce high-quality, well-designed studies with adequate follow-up, in order to evaluate all healthcare quality dimensions (including cost-efficacy and equity) of hospital mergers. In particular, quasi-experimental studies with interrupted time series design can evaluate intervention effect estimating causal effects using observational approaches when, as in this case, randomized controlled trials (the ideal approach to evaluate effects of interventions) cannot be performed.^37–39^ This robust design can be employed to understand the effects of policies and the improvement in the health system quality also thanks to the ability of controlling for the secular trends present in many health system outcomes^38^^,^^39^ (available in the Supplementary data). Policy and decision makers and hospital managers should take into account that a merger should not start without a clear vision and continuous appraisal of benefits, drawbacks and the expected impact of the merger on patients and staff, in an evidence-based policymaking framework that helps to perform an overall periodic assessment of processes and outcomes and to adopt the appropriate corrective measures, whenever necessary.

## Supplementary data

Supplementary data are available at *EURPUB* online.

## Funding 

None.

*Conflicts of interest*: None declared.

Key points

- In the last decades, an increasing number of hospitals have undergone a merger but literature on the impact of merger on hospitals Health Quality Measures (HQM) is still lacking.
- Studies included in our systematic review resulted to be heterogeneous in HQM analyzed and methodology adopted mainly providing only descriptive statistics and showing moderate to low risk of bias, few statistically significant results and inconsistent findings across them.
- Additional high-quality and well-designed studies with adequate follow-up are needed ascertaining mergers do not reduce the quality of care.
- Policymakers and hospital managers who are going to start a merger process must early adopt an evaluation framework that helps to perform an overall periodic assessment of HQM during the whole process.

## Supplementary Material

ckac002_Supplementary_Data
