# Supplementary material for: Impact of hospital mergers: a systematic review focusing on healthcare quality measures
Source: Eur J Public Health. 2022 Feb 14;32(2):191–9. doi: 10.1093/eurpub/ckac002 (PMC9090279; doi:10.1093/eurpub/ckac002)
Supplement: ckac002_Supplementary_Data [file ckac002_supplementary_data.docx]

**Supplementary information.**

**Search String by databases.**

PUBMED: ("hospitals"[All Fields] OR "hospital"[All Fields]) AND ("Health Facility Merger"[Mesh] OR "merger"[All Fields] OR "mergers"[All Fields] OR "merged"[All Fields] OR "merging"[All Fields] OR "consolidation"[All Fields] OR "consolidations"[All Fields]) AND ((("Outcome"[All Fields] OR "Process"[All Fields] OR "Outcomes"[All Fields] OR "Processes"[All Fields]) AND ("indicators"[All Fields] OR "indicator"[All Fields] OR "measure"[All Fields] OR "measures"[All Fields]) OR "performance"[All Fields]))

SCOPUS: (("hospital" AND ("merger" OR "mergers" OR "merged" OR "merging" OR “consolidation” OR “acquisition” OR “acquisitions”) AND (("Outcome" OR "Process") AND ("indicators" OR "indicator" OR “measure” OR “measures”) OR "performance")) - search restricted to TITLE/ABSTRACT/KEYWORDS

WEB OF SCIENCE: ("hospitals" OR "hospital") AND ("merger" OR "mergers" OR "merged" OR "merging" OR “consolidation” OR “consolidations” OR “acquisition” OR “acquisitions” ) AND ("Outcome" OR "Process" OR "indicators" OR "indicator" OR “measure” OR “measures” OR "performance") - search restricted to TITLE/ABSTRACT/KEYWORDS

**Supplementary Table 1. Overall quality assessment of single studies.**

| **Author, Year** | **Quality assessment** |
| --- | --- |
| **Before-after studies** | |
| Alexander, 1997 | Low risk |
| Beaulieu, 2020 | Low risk |
| Dranove, 2002 | Low risk |
| Gaynor, 2012 | Low risk |
| Harris, 2000 | Low risk |
| Hayford, 2012 | Low risk |
| Ho, 2000 | Low risk |
| Ingebrigtsen, 2012 | Moderate risk |
| Romano, 2010 | Moderate risk |
| Christiansen, 2018 | Moderate risk |
| **Case-control studies** | |
| Alexander, 1996 | Low risk |
| Beaulieu, 2020 | Low risk |
| Romano, 2010 | Low risk |
| Dranove, 2002 | Low risk |
| Gaynor, 2012 | Low risk |
| **Cross-sectional studies on perception/satisfaction of hospital staff** | |
| Engstrom, 2002 | Moderate risk |
| Holm-Peterson, 2017 | Low risk |
| Roald, 2001 | Moderate risk |
| Shaw, 2002 | Moderate risk |
| Stankova, 2018 | Moderate risk |
| Noether, 2017 | Moderate risk |

**Additional references:**

1. Tugwell P, Knottnerus JA, McGowan J, et al. Big-5 quasi-experimental designs. 89, 2017: 1–3.
2. Victora CG, Habicht J-P, Bryce J. Evidence-Based public health: moving beyond randomized trials. Am J Public Health 2004;94:400–5.doi:10.2105/AJPH.94.3.400pmid:http://www.ncbi.nlm.nih.gov/pubmed/14998803
3. Black N. Why we need observational studies to evaluate the effectiveness of health care. BMJ1996;312:12158.doi:10.1136/bmj.312.7040.1215pmid:http://www.ncbi.nlm.nih.gov/pubmed/8634569
4. Penfold RB, Zhang F. Use of interrupted time series analysis in evaluating health care quality improvements. Acad Pediatr 2013;13:S38–44.doi:10.1016/j.acap.2013.08.002pmid:http://www.ncbi.nlm.nih.gov/pubmed/24268083
5. Fretheim A, Tomic O. Statistical process control and interrupted time series: a golden opportunity for impact evaluation in quality improvement. BMJ Qual Saf 2015;24:748–52.doi:10.1136/bmjqs-2014-003756pmid:http://www.ncbi.nlm.nih.gov/pubmed/26316541
6. Lagarde M. How to do (or not to do). Assessing the impact of a policy change with routine longitudinal data. Health Policy Plan 2012;27:76–83.doi:10.1093/heapol/czr004pmid:http://www.ncbi.nlm.nih.gov/pubmed/21278077
